# Reciprocal intern‐nurse shadowing program may lead to improved interprofessional collaboration

**DOI:** 10.1002/jhm.70168

**Published:** 2025-10-26

**Authors:** Lauren S. Starnes, Joseph R. Starnes, Beth Loats, Cristina Loaiza, Vicki Jones, Alison Herndon

**Affiliations:** ^1^ Vanderbilt University Medical Center Nashville Tennessee USA

## Abstract

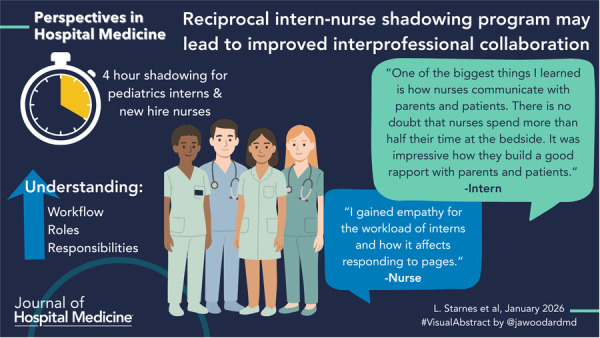

## INTRODUCTION

Positive nurse–physician collaboration improves outcomes and patient‐centered care.^1^, ^2^, ^3^ Developing trust, communication skills, respect, and role understanding promotes collaboration.^2^ Challenges to collaboration include individual experiences, hospital culture, and perceived provider hierarchy.^3^ Despite inclusion in core competencies for trainees, interprofessional collaboration is insufficiently prioritized in medical education.^2^ Physicians and nurses are trained separately, lack a shared communication framework,^4^ and have few opportunities for intentional interprofessional learning.^5^

Shadowing programs provide dedicated time to develop collaboration skills.^4^, ^6^, ^7^, ^8^, ^9^, ^10^ Current literature largely describes medical student programs, with limited examples involving pediatric residents. Further, few studies use reciprocal shadowing, in which both nurses and physicians shadow in the other profession.^6^, ^9^ Existing work has relied on participant self‐evaluation of knowledge, attitudes, or competency as outcomes.^7^ Additionally, current literature fails to describe the educational framework used to develop programs; others have also argued that programs have poor methodological design.^7^ We developed a reciprocal shadowing program between pediatric interns and acute care inpatient pediatric nurses rooted in social cognitive theory.^11^ We aimed to improve participants’ knowledge of the roles and workflow of the other profession, attitudes toward collaboration, and perceived interprofessional communication skills.

## THE INTERN‐NURSE SHADOWING PROGRAM

We designed a mixed‐methods study including a 4‐h shadowing experience in which interns and newly hired nurses observed the other profession at a 343‐bed tertiary care facility from January 2020 to June 2023. We required all pediatric interns to shadow a nurse during an afternoon on their hospital medicine rotation. Interns shadowed nurses who had worked on the acute care unit for at least 6 months and volunteered to work with trainees. We also required all new‐hire nurses to shadow an intern during an orientation afternoon.

Key components of curricula designed using social cognitive theory include *clear learning objectives, foundation of task‐relevant knowledge, modelling, guided practice, and reflection*.^12^ Participants received *learning objectives* for the shadowing experience (File S1). Shadows and hosts also received a guideline for the shadowing experience, which included questions to discuss to solidify *foundational knowledge* about the other profession (aside from any *foundational knowledge* they may have already acquired through their training) and recommendations on activities to observe (*modelling*) and in which to participate (*guided practice*) (File S1). We created the guideline through iterative discussion with our interprofessional study team, which included pediatric residents, the acute care unit medical director, the associate officer of inpatient nursing services, and inpatient nursing managers. We aimed to focus on areas in which residents and nurses expressed frustrations during routine feedback meetings.

Shadows completed an online, anonymous Research Electronic Data Capture (REDCap) survey before participating.^13^, ^14^ Surveys included multiple‐choice knowledge questions about the profession participants would shadow, statements with four‐point Likert‐type responses (1 = *strongly disagree*, 4 = *strongly agree*), and the validated Jefferson Scale of Attitudes toward Physician‐Nurse Collaboration (JSATPNC).^15^ Postshadowing surveys included the same content, as well as open‐ended, qualitative questions (encouraging *reflection*) and quantitative program feedback. We administered postshadowing surveys the day after shadowing and asked for completion within 2 weeks. Surveys are in File S2 **.** This study was deemed exempt by the Vanderbilt University Institutional Review Board (#210294).

### Analysis

We calculated the JSATPNC score as previously described.^15^ We used the Wilcoxon rank‐sum test and Fisher's exact test to compare preshadowing and postshadowing values. We performed all analyses using Stata version 14.2 (StataCorp LP). Two authors (L. S. S. and J. R. S.) inductively coded qualitative data. Authors iteratively discussed codes to develop themes and subthemes. The study group reviewed themes to reach a consensus.^16^

## QUANTITATIVE OUTCOMES OF THE PROGRAM

Fifty‐two nurses (100%) and 60 interns (89.5%) participated. Of participants, 48 (92%) nurses and 53 (88%) interns completed presurveys, and 34 (65%) nurses and 28 (47%) interns completed postsurveys.

### Knowledge and understanding

After shadowing, interns had more correct responses to the average days per week nurses work (preshadowing correct: 26% vs. postshadowing correct: 57%, *p* = .007) and a nonsignificant trend toward more correct responses to nursing morning (98% vs. 100%) and evening sign out time (92% vs. 96%). Nurses had more correct responses to intern morning (29% vs. 79%, *p* < .001) and evening sign‐out time (8% vs. 32%, *p* = .008). We found no correct responses before or after shadowing for the average number of days per week that interns work.

Interns were more likely to respond that they understood the daily workflow of nurses after shadowing (*p* < .001). We also saw a trend in responses suggesting they better understood the responsibilities of nurses (*p* = .0568) but not nursing training (*p* = .9909). Nurses reported better understanding interns’ responsibilities, workflow, and training (all *p* < .001).

### JSATPNC scores

The maximum possible score on the JSATPNC is 60, with higher scores indicating more favorable attitudes toward collaboration. Intern median preshadowing score was 54.5 (inter‐quartile range 47.5, 56) compared with 52 (47, 57) postshadowing. Nurse median preshadowing score was 53 (50.5, 56.5) compared with 54 (52, 56) postshadowing. There was no significant difference between pre‐ and postshadowing scores for either interns or nurses (*p* = .69 and.80, respectively).

### Perceived impact of program

The majority of interns (25, 89.2%) and nurses (31, 91.2%) agreed/strongly agreed that the program changed their daily practice. They also felt shadowing made them more effective at interprofessional teamwork (interns: 24, 85.7%; nurses: 34, 100%) and communication (interns: 20, 71.4%; nurses: 34, 100%).

## QUALITATIVE OUTCOMES OF THE PROGRAM

Major themes from participant reflection and feedback about program strengths included: *communication, relationships, process understanding, collaboration, and practice transformation*. *Communication* was the most frequently identified theme. Many reported improved ability to communicate with the profession they shadowed through learning how they prefer to be communicated with and desired information. The experience helped participants recognize the importance of interprofessional communication. Many nurses reported improved comfort with speaking with physicians. *Relationships* were another major theme in responses. Many respondents felt the experience provided dedicated time to talk with an individual in the other profession and build a relationship. Several felt shadowing engendered trust and humanized individuals in the profession observed. Participants responded with empathy toward the other profession, especially in reference to pages.

Participants also gained a better understanding of *processes* involved in patient care. Both interns and nurses felt that shadowing helped them better understand the workflow, roles, and responsibilities of the professionals they shadowed and that this understanding led to respect. Interns felt that shadowing allowed them to learn more about the barriers and challenges nurses face and specifically commented that they have more appreciation for how long nursing tasks might take and how their orders affect nurses’ ability to provide care. Additionally, participants reported that the experience reinforced the value of and their skills in *interprofessional collaboration*. Several interns commented on tangible ways that shadowing would *change their practice*. Many of these revolved around how and when orders are placed, and an appreciation for the fact that a nurse is responsible for executing those orders. One intern also commented that they identified a patient safety event that resulted in a change of practice across the hospital. Themes, subthemes, and example quotes can be found in Table 1.

**Table 1:** Themes and example quotes from open‐ended responses.

Theme	Subtheme	Example quotes
Communication	Improved interprofessional communication	Intern: “I liked being able to talk to the nurses and ask what they found helpful and what they did not find helpful with respect to communication.” Nurse: “We talked about the amount of pages that interns get in a day, and I asked her what is most helpful when sending pages. I learned to be more detailed with my concerns in the page.”
	Importance of communication	Intern: “I realize how frustrating I can make their job with poor communication, and conversely, how much more smoothly it goes with clear direction and communication.” Nurse: “It made me realize how important it is for patients’ safety and course of care to be good communicators.”
	Improved comfort with communication	Nurse: “Shadowing the intern really helped with diminishing that fear new nurses can have of communicating with physicians as part of the interdisciplinary team.” Nurse: “It made me more confident to speak with members of the team by getting to know a few of the names I see assigned to my patients for a few hours. I feel less intimidated and more prepared and comfortable collaborating with the physicians assigned to my patients.”
Relationships	Built meaningful relationships	Nurse: “I think it helped create a more personal relationship with the interns.” Intern: “Building stronger personal relationships with the nurses I worked with improves our joint patient care moving forward.” Intern: “I feel like if we get to know one another a little more personally then we can communicate better.”
	Engendered trust	Nurse: “It made me feel good that now they trust my opinion, or that I may have the answer for them. I feel like if they get to know us better and we're paging them, and we're concerned, they'll be concerned too.”
	Humanized the other profession	Intern: “Getting to know the nurse I shadowed was so fun. We're now friends on [social media].” Intern: “When you work together, you see each other as human.”
	Created empathy for workload	Nurse: “I gained empathy for the workload of interns and how it affects responding to pages.”
Process understanding	Helped with role understanding	Intern: “One of the biggest things I learned is how nurses communicate with parents and patients. There is no doubt that nurses spend more than half their time at the bedside. It was impressive how they build a good rapport with parents and patients.”
	Helped with workflow understanding	Nurse: “It was very good to not only see the intern interacting with the patients but seeing what she had to do immediately after as well. As a nurse, we often see the physician in the room, but rarely see what happens immediately before and after that event in the physician's workflow.”
	Identified challenges to patient care	Intern: “The other thing that surprised me is how long it takes for meds to come up from the pharmacy, and that's a reason why patients get medications late.”
Collaboration	Reinforced value of interprofessional collaboration	Intern: “I have always valued interprofessional collaboration, however, this has strengthened it. It would be interesting to do this with other care providers as well.”
	Improved ability to collaborate	Nurse: “I think it affected my ability to collaborate efficiently with physicians in the future.”
Practice Transformation	Changed daily practice	Intern: “I am more respectful of their sign out time. I'll order labs a little earlier knowing that they can take longer. And I try to bunch medications at the same time.”
	Identified patient safety issues	Intern: “While shadowing a nurse, we talked about the discharge contingency program. From a nursing perspective, the order was not displaying with a contingency until it was clicked on multiple times. We discovered that this was not safe for patients (and no one's fault given the display of the order). I emailed the Chief Residents who escalated the problem, eventually reaching the Chief of Staff. The feedback resulted in a system‐wide change, and the order was removed. I would have never known about this if it weren't for this experience!”

Interns noted that it could be difficult to leave their rotation and suggested housing the program within an outpatient rotation. Nurses also requested to shadow during morning rounds. Respondents in both groups stated that shadowing would be beneficial for individuals in the other profession to participate in as well, unaware that the program was reciprocal.

## RECOMMENDATIONS AND NEXT STEPS

We demonstrated that a shadowing program rooted in social cognitive theory improved participants’ knowledge of the other profession's workflow and roles, improved their interprofessional teamwork and communication, built relationships, engendered trust and empathy, reinforced the value of interprofessional collaboration, and identified changes in practice. These overlap with analyses of other shadowing programs,^4^, ^9^, ^10^, ^17^ further supporting the efficacy of this educational modality. We suggest that physicians and nurses engage in interprofessional shadowing at the start of their hiring to develop these skills early.

There are limited studies evaluating the inclusion of both interns and nurses in shadowing. Our study adds to the literature on reciprocal programs, supporting findings that this format impacts participants’ self‐perceived attitudes and competence.^6^, ^9^, ^10^ We also evaluated changes in participants’ knowledge about the other profession and their workflow, which is unique from other shadowing program studies.^4^, ^6^, ^7^, ^9^, ^18^ Interns had more correct responses about nursing schedules after shadowing. Nurses showed improved responses in intern sign‐out times. Nursing responses on the average number of days that interns work both before and after shadowing were incorrect. In reviewing responses, almost all chose 5 days per week after shadowing, whereas the range before shadowing was 2–5 days. Although interns work 6 days per week on their hospital medicine rotation, these responses still suggest increased understanding of the work schedule of interns.

After shadowing, interns reported improved understanding of nursing workflow and had nonsignificant trend in improved understanding of nursing responsibilities. Our decrease in postsurvey responses may have led to our inability to detect a significant change. Interns’ self‐reported understanding of nursing training was unchanged, which aligns with our findings on knowledge questions. Nurses had an improved understanding of interns’ workflow, responsibilities, and training. Combined, these findings support that our program effectively improved participants’ knowledge about the other profession.

We did not find a significant change in responses to the JSATPNC in alignment with other studies that used this tool.^2^, ^6^ Median scores before shadowing were high, and it may be that we did not observe a change due to the ceiling effect.^19^ The JSATPNC may not be sensitive to change in this setting.

In reviewing suggested opportunities for improvement, our interns now shadow after a morning clinic. A potential barrier to sustainability of our program is time within resident and nursing schedules, in addition to having hospital staff with the capacity to organize logistics of the program. Additionally, both interns and nurses commented that they felt it would be important for the other profession to shadow. This highlighted our need to make participants aware of the reciprocal nature of our shadowing program and advertise the program more broadly in our hospital system.

Our study is limited by program implementation at a single tertiary care institution but may be generalizable to similar hospital systems or training programs. Additionally, we did not collect data from hosts, which would help us understand how this program impacts those in that role. We were unable to pair presurvey and postsurvey responses in our study and had attrition in responses to our postsurvey. It may be that participants who viewed the experience positively were more likely to respond to our survey, which may have skewed our results. Finally, we did not evaluate effects on behavior and patient care. Future work should further explore the potential for our program to impact patient care.

## CONFLICT OF INTEREST STATEMENT

The authors declare no conflicts of interest.

## Supporting information

INSP Supplements.
