# Supplementary material for: Reciprocal intern‐nurse shadowing program may lead to improved interprofessional collaboration
Source: J Hosp Med. 2025 Oct 26;21(1):80–4. doi: 10.1002/jhm.70168 (PMC12747489; doi:10.1002/jhm.70168)
Supplement: Supplementary file 1 — INSP Supplements. [file JHM-21-80-s001.docx]

**Supplement 1: Guidelines for Shadowing**

**Guideline for Intern Shadowing Nurse**

**Learning Objectives**

By the end of this session, you will be able to:

- Describe the roles and responsibilities of the healthcare professional you shadow
- Define the daily workflow of the healthcare professional you shadow
- Describe the training required for the healthcare professional you shadow
- Analyze communication between nurses and interns and consider opportunities for improvement
- Recognize that interprofessional collaboration is important for providing high-quality patient care

**Introductions**

Consider asking the nurse you are paired with the following questions:

- Can you tell me about being a nurse?
- What are your career interests?
- How many years of training do you need to complete?

**Information Gathering**

Consider asking the nurse you are paired with the following questions:

- Can you discuss your daily workflow?
- About how many days will you work in a week?
- About how many patients do you carry?
- What quality improvement measures affect your work?

**Observation**

You will observe the nurse throughout the afternoon. Consider taking note of the following:

- How long does it take nurses to complete their charting?
- When are the busiest times of the day?
- How often does the nurse receive pages?
- How does the nurse prefer to communicate with interns?
- Are there examples of effective or ineffective communication with interns?

**Participation**

Please participate in at least three of the following activities:

- Admit a patient
- Discharge a patient
- Assess a patient
- Administer medication
- Administer fluids
- Administer NG tube or G-tube feeds
- Manage an IV and/or line
- Collect blood or specimen
- Observe report

**Closing**

Consider asking the nurse you are paired with the following questions:

- Did I observe a typical day for you?
- What do you enjoy most about your job?
- What are the top 1-2 challenges of your job?
- How can interns and nurses communicate more effectively?

**Guideline for Nurse Shadowing Intern**

**Learning Objectives**

By the end of this session, you will be able to:

- Describe the roles and responsibilities of the healthcare professional you shadow
- Define the daily workflow of the healthcare professional you shadow
- Describe the training required for the healthcare professional you shadow
- Analyze communication between nurses and interns and consider opportunities for improvement
- Recognize that interprofessional collaboration is important for providing high-quality patient care

**Introductions**

Consider asking the intern you are paired with the following questions:

- Can you tell me about being a resident?
- What are your career interests?
- How many years of training do you need to complete?

**Information Gathering**

Consider asking the intern you are paired with the following questions:

- Can you discuss your daily workflow?
- About how many days will you work in a week?
- About how many patients are typically on your team, and how do you divide them?
- What quality improvement measures affect your work?

**Observation**

You will observe the intern throughout the afternoon. Consider taking note of the following:

- How many notes does the intern write and how long does it take to complete them?
- When are the busiest times of the day?
- How often does the intern receive pages?
- How does the intern prefer to communicate with nurses?
- Are there examples of effective or ineffective communication with nurses?
- How does the intern communicate with patients?

**Participation**

Please participate in at least three of the following activities:

- Admit a patient
- Discharge a patient
- Assess a patient
- Place orders for medications, fluids, and/or tube feeds
- Place orders for tests and/or procedures
- Discuss a patient with a consultant
- Participate in afternoon teaching sessions
- Run the list with the team
- Sign out of a patient

**Closing**

Consider asking the intern you are paired with the following questions:

- Did I observe a typical day for you?
- What do you enjoy most about your job?
- What are the top 1-2 challenges of your job?
- How can interns and nurses communicate more effectively?

**Supplement 2: Study Surveys**

**Intern Pre-Shadowing Survey**

**Knowledge Section**

1. On average, how many days per week do nurses work?
   1. 2
   2. 3
   3. 4
   4. 5
2. What time is nursing sign out in the morning?
3. 0500
4. 0600
5. 0700
6. 0800
7. What time is nursing sign out at night?
8. 1700
9. 1800
10. 1900
11. 2000
12. How many years of schooling is required for a BSN?
13. 2
14. 3
15. 4
16. 5

**Jefferson Scale of Attitudes Towards Physician-Nurse Collaboration**

*On a scale from 1 = strongly disagree to 4 = strongly agree, please rate your agreement with the following statements:*

1. Physicians should be educated to establish collaborative relationships with nurses.
2. Interprofessional relationships between physicians and nurses should be included in their educational programs.
3. Nurses should be involved in making policy decisions concerning the hospital support services upon which their work depends.
4. Nurses should also have responsibility for monitoring the effects of medical treatment.
5. Nurses should clarify a physician’s order when they feel that it might have the potential for detrimental effects on the patient.
6. Nurses should be involved in making policy decisions affecting their working conditions.
7. A nurse should be viewed as a collaborator and colleague with a physician rather than his/her assistant.
8. Nurses are qualified to assess and respond to psychological aspects of patients’ needs.
9. Nurses have special expertise in patient education and psychological counseling.
10. Nurses should be accountable to patients for the nursing care they provide.
11. During their education, medical and nursing students should be involved in teamwork in order to understand their respective roles.
12. Physicians and nurses should contribute to decisions regarding the hospital discharge of patients.
13. There are many overlapping areas of responsibility between physicians and nurses.
14. Doctors should be the dominant authority in all health care matters.
15. The primary function of the nurse is to carry out the physician’s orders.

**Additional Questions**

*On a scale from 1 = strongly disagree to 4 = strongly agree, please rate your agreement with the following statements:*

1. I understand the responsibilities of nurses.
2. I understand the daily workflow of nurses.
3. I understand the training required to be a nurse.

**Nurse Pre-Shadowing Survey**

**Knowledge Section**

1. On average, how many days per week do interns work?
2. 4
3. 5
4. 6
5. 7
6. What time is intern sign out in the morning?
7. 0500
8. 0600
9. 0700
10. 0800
11. What time is intern sign out at night?
12. 1700
13. 1800
14. 1900
15. 2000
16. How many years is a pediatric residency?
17. 2
18. 3
19. 4
20. 5

**Jefferson Survey of Attitudes Towards Physician-Nurse Collaboration**

*On a scale from 1 = strongly disagree to 4 = strongly agree, please rate your agreement with the following statements:*

1. Physicians should be educated to establish collaborative relationships with nurses.
2. Interprofessional relationships between physicians and nurses should be included in their educational programs.
3. Nurses should be involved in making policy decisions concerning the hospital support services upon which their work depends.
4. Nurses should also have responsibility for monitoring the effects of medical treatment.
5. Nurses should clarify a physician’s order when they feel that it might have the potential for detrimental effects on the patient.
6. Nurses should be involved in making policy decisions affecting their working conditions.
7. A nurse should be viewed as a collaborator and colleague with a physician rather than his/her assistant.
8. Nurses are qualified to assess and respond to psychological aspects of patients’ needs.
9. Nurses have special expertise in patient education and psychological counseling.
10. Nurses should be accountable to patients for the nursing care they provide.
11. During their education, medical and nursing students should be involved in teamwork in order to understand their respective roles.
12. Physicians and nurses should contribute to decisions regarding the hospital discharge of patients.
13. There are many overlapping areas of responsibility between physicians and nurses.
14. Doctors should be the dominant authority in all health care matters.
15. The primary function of the nurse is to carry out the physician’s orders.

**Additional Questions**

*On a scale from 1 = strongly disagree to 4 = strongly agree, please rate your agreement with the following statements:*

1. I understand the responsibilities of interns.
2. I understand the daily workflow of interns.
3. I understand the training required to be a physician.

**Intern Post-Shadowing Survey**

**Knowledge Section**

1. On average, how many days per week do nurses work?
2. 2
3. 3
4. 4
5. 5
6. What time is nursing sign out in the morning?
7. 0500
8. 0600
9. 0700
10. 0800
11. What time is nursing sign out at night?
12. 1700
13. 1800
14. 1900
15. 2000
16. How many years of schooling is required for a BSN?
17. 2
18. 3
19. 4
20. 5

**Jefferson Survey of Attitudes Towards Physician-Nurse Collaboration**

*On a scale from 1 = strongly disagree to 4 = strongly agree, please rate your agreement with the following statements:*

1. Physicians should be educated to establish collaborative relationships with nurses.
2. Interprofessional relationships between physicians and nurses should be included in their educational programs.
3. Nurses should be involved in making policy decisions concerning the hospital support services upon which their work depends.
4. Nurses should also have responsibility for monitoring the effects of medical treatment.
5. Nurses should clarify a physician’s order when they feel that it might have the potential for detrimental effects on the patient.
6. Nurses should be involved in making policy decisions affecting their working conditions.
7. A nurse should be viewed as a collaborator and colleague with a physician rather than his/her assistant.
8. Nurses are qualified to assess and respond to psychological aspects of patients’ needs.
9. Nurses have special expertise in patient education and psychological counseling.
10. Nurses should be accountable to patients for the nursing care they provide.
11. During their education, medical and nursing students should be involved in teamwork in order to understand their respective roles.
12. Physicians and nurses should contribute to decisions regarding the hospital discharge of patients.
13. There are many overlapping areas of responsibility between physicians and nurses.
14. Doctors should be the dominant authority in all health care matters.
15. The primary function of the nurse is to carry out the physician’s orders.

**Additional Questions**

*On a scale from 1 = strongly disagree to 4 = strongly agree, please rate your agreement with the following statements:*

1. I understand the responsibilities of nurses.
2. I understand the daily workflow of nurses.
3. I understand the training required to be a nurse.
4. This experience improved my interprofessional communication skills.
5. I will be a more effective member of an interprofessional team because of this experience.
6. This experience will change my daily practice.

**Nurse Post-Shadowing Survey**

**Knowledge Section**

1. On average, how many days per week do interns work?
2. 4
3. 5
4. 6
5. 7
6. What time is intern sign out in the morning?
7. 0500
8. 0600
9. 0700
10. 0800
11. What time is intern sign out at night?
12. 1700
13. 1800
14. 1900
15. 2000
16. How many years is a pediatric residency?
17. 2
18. 3
19. 4
20. 5

**Jefferson Survey of Attitudes Towards Physician-Nurse Collaboration**

*On a scale from 1 = strongly disagree to 4 = strongly agree, please rate your agreement with the following statements:*

1. Physicians should be educated to establish collaborative relationships with nurses.
2. Interprofessional relationships between physicians and nurses should be included in their educational programs.
3. Nurses should be involved in making policy decisions concerning the hospital support services upon which their work depends.
4. Nurses should also have responsibility for monitoring the effects of medical treatment.
5. Nurses should clarify a physician’s order when they feel that it might have the potential for detrimental effects on the patient.
6. Nurses should be involved in making policy decisions affecting their working conditions.
7. A nurse should be viewed as a collaborator and colleague with a physician rather than his/her assistant.
8. Nurses are qualified to assess and respond to psychological aspects of patients’ needs.
9. Nurses have special expertise in patient education and psychological counseling.
10. Nurses should be accountable to patients for the nursing care they provide.
11. During their education, medical and nursing students should be involved in teamwork in order to understand their respective roles.
12. Physicians and nurses should contribute to decisions regarding the hospital discharge of patients.
13. There are many overlapping areas of responsibility between physicians and nurses.
14. Doctors should be the dominant authority in all health care matters.
15. The primary function of the nurse is to carry out the physician’s orders.

**Additional Questions**

*On a scale from 1 = strongly disagree to 4 = strongly agree, please rate your agreement with the following statements:*

1. I understand the responsibilities of interns.
2. I understand the daily workflow of interns.
3. I understand the training required to be a physician.
4. This experience improved my interprofessional communication skills.
5. I will be a more effective member of an interprofessional team because of this experience.
6. This experience will change my daily practice.
